# Differential effects of garcinol and curcumin on histone and p53 modifications in tumour cells

**DOI:** 10.1186/1471-2407-13-37

**Published:** 2013-01-29

**Authors:** Hilary M Collins, Magdy K Abdelghany, Marie Messmer, Baigong Yue, Sian E Deeves, Karin B Kindle, Kempegowda Mantelingu, Akhmed Aslam, G Sebastiaan Winkler, Tapas K Kundu, David M Heery

**Affiliations:** 1 Gene Regulation Group, Centre for Biomolecular Sciences, School of Pharmacy, University of Nottingham, University Park, NG7 2RD, Nottingham, United Kingdom; 2 Transcription and Disease Laboratory, Jawaharlal Nehru Centre for Advanced Scientific Research, 560064, Bangalore, Karnataka, India; 3 Present address: Department of Pathology, Faculty of Medicine, Suez Canal University, Ismailia, Egypt

**Keywords:** Garcinol, Curcumin, Acetyltransferase, HAT inhibitor, Histones, p53, Post-translational modifications, H4K20Me3, SUV420H2, TIP60

## Abstract

**Background:**

Post-translational modifications (PTMs) of histones and other proteins are perturbed in tumours. For example, reduced levels of acetylated H4K16 and trimethylated H4K20 are associated with high tumour grade and poor survival in breast cancer. Drug-like molecules that can reprogram selected histone PTMs in tumour cells are therefore of interest as potential cancer chemopreventive agents. In this study we assessed the effects of the phytocompounds garcinol and curcumin on histone and p53 modification in cancer cells, focussing on the breast tumour cell line MCF7.

**Methods:**

Cell viability/proliferation assays, cell cycle analysis by flow cytometry, immunodetection of specific histone and p53 acetylation marks, western blotting, siRNA and RT-qPCR.

**Results:**

Although treatment with curcumin, garcinol or the garcinol derivative LTK-14 hampered MCF7 cell proliferation, differential effects of these compounds on histone modifications were observed. Garcinol treatment resulted in a strong reduction in H3K18 acetylation, which is required for S phase progression. Similar effects of garcinol on H3K18 acetylation were observed in the osteosarcoma cells lines U2OS and SaOS2. In contrast, global levels of acetylated H4K16 and trimethylated H4K20 in MCF7 cells were elevated after garcinol treatment. This was accompanied by upregulation of DNA damage signalling markers such as γH2A.X, H3K56Ac, p53 and TIP60. In contrast, exposure of MCF7 cells to curcumin resulted in increased global levels of acetylated H3K18 and H4K16, and was less effective in inducing DNA damage markers. In addition to its effects on histone modifications, garcinol was found to block CBP/p300-mediated acetylation of the C-terminal activation domain of p53, but resulted in enhanced acetylation of p53K120, and accumulation of p53 in the cytoplasmic compartment. Finally, we show that the elevation of H4K20Me3 levels by garcinol correlated with increased expression of SUV420H2, and was prevented by siRNA targeting of SUV420H2.

**Conclusion:**

In summary, although garcinol and curcumin can both inhibit histone acetyltransferase activities, our results show that these compounds have differential effects on cancer cells in culture. Garcinol treatment alters expression of chromatin modifying enzymes in MCF7 cells, resulting in reprogramming of key histone and p53 PTMs and growth arrest, underscoring its potential as a cancer chemopreventive agent.

## Background

Enzymes that modify chromatin and associated proteins by the addition or removal of acetyl or methyl groups play a key role in genome regulation [1]. These and other PTMs generate a combinatorial histone code that demarcates chromatin regions for transcription activation or repression [2]. Histone PTMs are also critical for other genomic functions, such as DNA replication and induction of repair mechanisms at sites of DNA damage [3]. Histone modifications act as signals that are ‘read’ by sensory proteins containing bromodomains, PHD fingers and other domains, many of which function as coregulators of DNA-binding transcription factors [4]. While some core histone PTMs (H3K9Ac, H3K18Ac, H3K27Ac, H3K4Me3) are commonly associated with active genes, others (H3K9Me2/3 and H4K20Me3) are more usually indicators of repressed genes and heterochromatin [1,2]. Specific histone PTMs such as phospho-S139H2AX (known as γH2A.X) are upregulated by DNA damage signalling, and are necessary for DNA repair [3,5]. Not surprisingly, dramatic changes in global histone PTMs are observed in cancer, such as the observed reduction of H4K16Ac and H4K20Me3 levels in cancer cell lines, described as hallmarks of cancer [6]. This was recently confirmed in tissue microarray studies using large numbers of breast and prostate tumours [7-10]. Interestingly, H4K16Ac and H4K20Me3 are also implicated in DNA damage checkpoints [11,12] which are disrupted in cancer cells. Thus, drug-like molecules that target chromatin modifying enzymes to reprogram selected histone PTMs in tumour cells may have potential as cancer chemopreventive agents.

A number of natural and synthetic molecules that inhibit histone acetyltransferase (HAT) or histone deacetylase (HDAC) activities have been described. HDAC inhibitors have shown promise in clinical trials as anticancer therapies, especially when used in combination with other chemotherapies. Less is known regarding the *in vivo* effects of molecules that can inhibit lysine acetyltransferase activity *in vitro*. Natural products that can block the activity of histone acetyltransferases *in vitro* have been isolated from plants [13-15]. Curcumin (diferuloylmethane) is derived from the turmeric plant *Curcuma longa* and inhibits CBP/p300 acetyltransferase activity *in vitro*, whereas PCAF appears insensitive to this compound at concentrations that inhibit p300 [16]. Garcinol is a polyisoprenylated benzophenone present in *Garcinia indica* fruit rind that also inhibits both CBP/p300 and PCAF HAT activities [17]. In this study we report that garcinol treatment blocks MCF7 cell proliferation, which is accompanied by induction of DNA damage repair markers and altered expression of selected histone/p53 modifying enzymes. This results in reprogramming of selected histone and p53 PTMs, and in particular can reverse the loss of H4K20Me3 in tumour cell lines. Our results provide insight into the biological effects of garcinol in altering histones and p53 PTMs in cancer cells, thus underscoring its potential as a lead for the development of new anticancer agents.

## Methods

### Acetyltransferase inhibitors

Curcumin was purchased from Sigma (C-1386). Garcinol was extracted as described previously [17], and LTK14 was synthesised from garcinol as previously described [18]. Inhibitor compounds were dissolved in DMSO (garcinol compounds) or ethanol (curcumin).

### Cell culture

The breast cancer cell line MCF7, and the osteosarcoma cell lines U2OS and SaOS2 were maintained in Dulbecco’s Modified Eagle Medium (DMEM) supplemented with 10% foetal calf serum (FCS) and 2 mM glutamine at 37°C in 5% CO_2_.

### Cell viability/proliferation assays

Viable cells were quantified by a standard MTT (3-(4,5-dimethylthiazol-2-yl)-2,5-di phenyltetrazolium bromide) reduction assay. Cell-mediated reduction of MTT was determined by reading absorbance at 550 nm. To measure the effects of curcumin, garcinol and LTK14 on cell viability and proliferation, MCF7 cells were seeded into 96-well microtitre plates at a density of 5 × 10^3^ cells/per well and allowed to adhere overnight. The initial density of viable cells prior to addition of inhibitors (denoted as time t=0) was determined in a control plate. Inhibitors were prepared immediately before use and added to test wells at the following concentrations (0, 2, 8, 15, 20 μM) at time zero. After addition of inhibitors or vehicle, cells were cultured for a further 24 hrs before measurement of MTT activities. Data were presented as the average of 5 replicates per condition.

### Western blots and immunocytochemistry

For western blotting and immuno-cytochemistry cells were cultured in DMEM supplemented with 10% FCS and 2 mM glutamine at 37°C in 5% CO_2,_ in the presence or absence of HAT inhibitors for 24 hours. Histones were acid extracted as described [19] for use in western blotting. For immunocytochemical detection of specific proteins or PTMs, cells were plated onto coverslips in 24 well plates for 24 hours. Following incubation with the inhibitors or vehicle, the cells were fixed in 4% paraformaldehyde and permeabilised using 0.2% Triton X-100 followed by a PBS wash, blocking in 3% BSA, and addition of primary antibodies as follows: pan acetyl H3 1:100, pan acetyl H4 1:100, Phospho-Ser139 H2A.X 1:75 (Upstate); H4K16Ac 1:200 (Chemicon); H3K9Ac 1:1000, H3K18Ac 1:200, H4K20Me3 1:200, TIP60 1:1000, p53K120Ac 1:100, p53K386Ac 1:200, p53K373/382Ac 1:100 (Abcam); p53(D01) 1:100 (Santa Cruz). After 1 hour incubation, the cells were washed in PBS and incubated with an appropriate secondary antibody (1:500 dilution). Images were captured on a Zeiss LSM510 Meta confocal microscope.

For western blotting, the above antibodies were used at a dilution of 1:500. In addition, other primary antibodies were H3 1:2000 (Santa Cruz); hMOF 1:200, TIP60 1:500 (Genetex); SIRT1 1:100, SUV420H1/H2 1:100, H3K9Me3 1:500 (Abcam) and H3K56Ac 1:500 (Epitomics). Appropriate HRP-conjugated secondary antibodies were used at a dilution of 1:5000 (Santa Cruz).

### Flow cytometry

To assess the effects of HAT inhibitors on the cell cycle, cells were treated with inhibitors or vehicle for 24 hours as described for growth assays, followed by addition of 1 μM Bromodeoxyuridine (BrdU) for 2 hours prior to harvesting. Cells were fixed, incubated with propidium iodide to stain DNA and FITC-conjugated anti-BrdU antibody 3D4 (BD Pharmingen), and subjected to bivariate flow cytometry as described previously [20]. To quantify the numbers of cells scoring positive for immunodetection of histone PTMs or SUV420H2 following exposure to HAT inhibitors, treated cells and controls were treated with trypsin, washed three times in PBS and fixed in ice-cold 70% ethanol. The permeabilised cells were incubated with primary antibodies (H4K20Me3 1:100 and SUV420H1/H2 1:100) and appropriate Alexa Fluor-conjugated secondary antibodies, and DNA labelled using propidium iodide. Cells were washed and resuspended in PBS for flow cytometric analysis using a FacsAria (BD Biosciences). The total number of cell scanning events was limited to 4000. Appropriate negative controls included unstained cells, PI only, secondary antibody only.

### RNA interference

The following siRNA duplexes were used (Dharmacon Research); SUV420H2 (on-targetplus SMARTpool L-018622-02), and nontargeting control siRNA (SMARTpool D-001810-10). MCF7 cells were transfected with siRNA (5 nM) using INTERFERin (Polyplus) following the manufacturer's instructions. At 24 hours post transfection cells were treated with 20 μM garcinol and western blotting carried out as above for the PTM H4K20Me3. SUV420H2 transcripts in the siRNA treated cells were measured by RT-qPCR, which was carried out as described previously [20] using the following primers; Fwd 5’cgtgtccactcgtgcttg-3’; Rev 5’ctcagcagcccctcatct-3. GAPDH transcript levels were used as the reference gene.

## Results

### Acetyltransferase inhibitors arrest MCF7 cell proliferation

**Figure 1:**
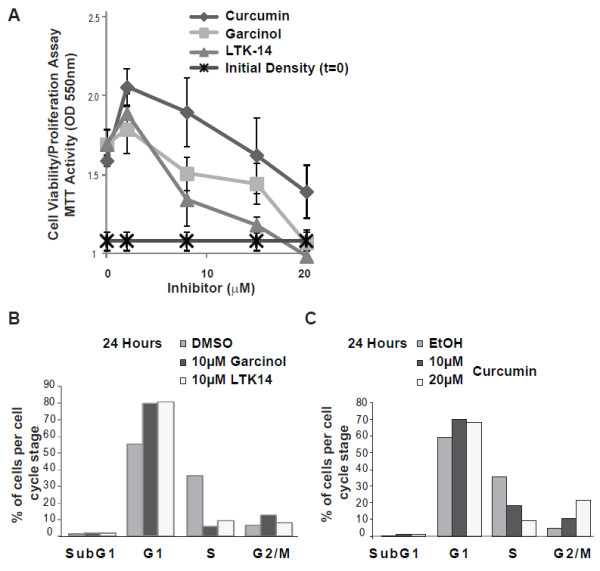
**Curcumin, garcinol and LTK-14 impede MCF7 cell proliferation. (A**) MCF7 cells were seeded at a density of approximately 5 × 10^3^ cells per well in microtitre plates and allowed to adhere overnight. The initial cell density was determined in a control plate prior to addition of curcumin, garcinol or LTK-14 at the indicated concentrations, or vehicle. After 24 hours, the change in the number of viable cells was estimated using MTT assays (see Methods). The data shown are the means of 5 replicates ± standard deviations. **(B)** Cell cycle analyses of MCF7 cells after 24 hours in culture in the presence of garcinol or LTK14 (10 μM) or vehicle control. The bar charts show a representative experiment indicating the percentage of cells in G1, S or G2/M phases, and the subG1 population as determined by BrdU incorporation and propidium iodide staining. **(C)** Cell cycle analyses of MCF7 cells after 24 hours culture in the presence of curcumin (10 μM or 20 μM) or vehicle control, as described in (**B**).

We assessed the effects of the acetyltransferase inhibitors curcumin and garcinol on proliferation of MCF7 cells in culture. Range finding experiments showed that concentrations above 20 μM of either compound were cytotoxic to MCF7 cells, inducing loss of adherence and cell lysis after 24 h exposure (data not shown). Thus, to facilitate the correlation of any antiproliferative effects with changes in histone modifications, cell proliferation/viability assays were performed using subcytotoxic concentrations of the HAT inhibitors. MCF7 cells were seeded at a density of 5 × 10^3^ cells/per well and allowed to adhere to plates overnight. MTT assays were performed to measure initial density of viable cells, and the change in cell viability/proliferation after culture for 24 h in the presence of a dose range (2-20 μM) of inhibitors or controls. The initial O.D. 550 nm readings (t=0) was 1.07 (shown as ‘initial density’ in Figure 1A) and after 24 hrs the control (vehicle-treated) cells showed an increase to an average reading of 1.67 O.D. units, indicative of an increase in viable cells due to proliferation. As shown in Figure 1A, in comparison to vehicle, curcumin had a stimulatory effect on the growth of MCF7 cells at the lowest dose (2 μM), but hampered cell proliferation at 20 μM. Inhibition of MCF7 cell growth by garcinol and LTK-14 was observed to be more potent, with a complete block of growth observed at 20 μM (Figure 1A). Similar results were obtained using U2OS cells (data not shown).

To confirm the observed effect of HAT inhibitor compounds on MCF7 cell growth, cell cycle analyses were performed using bivariate flow cytometry. As shown in Figure 1B, MCF7 cells exposed to garcinol (10 μM) for 24 hours showed a dramatic reduction in the numbers of actively replicating cells (S phase) compared to controls. This was accompanied by a concomitant increase in the G1 population, consistent with reduced proliferation and G1 arrest. Similar results were obtained using the recently described garcinol derivative 14-methoxy-isogarcinol (LTK-14) (Figure 1B) [18]. Thus, at subcytotoxic concentrations, garcinol compounds block the ability of MCF7 cells to successfully replicate their DNA.

Exposure of MCF7 cells to curcumin for 24 hours also resulted in a dose-dependent reduction in the proportion of cells entering S phase, as indicated in Figure 1C. In contrast to garcinol compounds, curcumin-treated MCF7 cells showed an increase of cells arrested in G2/M (a 4–fold increase over control after 24 hrs), (Figure 1C). Neither inhibitor induced a substantial increase in the sub-G1 population at the concentrations used, indicating that cells did not undergo apoptosis or cell death during the course of the experiment.

### Differential effects of curcumin and garcinol on histone PTMs

**Figure 2:**
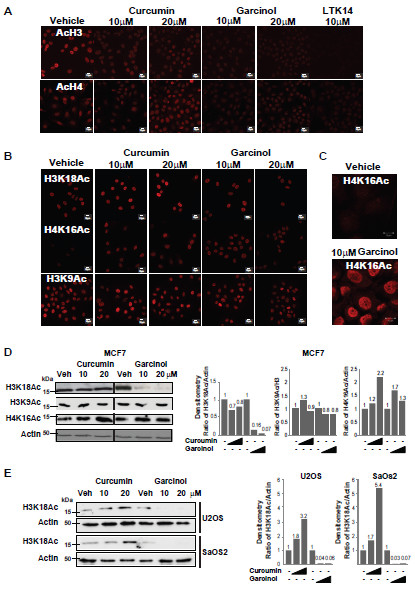
**Reprogramming of global histone modifications by garcinol and curcumin. (A)** Nuclear staining of MCF7 breast cancer cells with antibodies detecting pan acetyl H3 (top panels) or pan acetyl H4 (bottom panels). Control shows typical staining in the absence of inhibitors (vehicle only), and the effect of treatment with HAT inhibitors at the indicated concentrations are also shown. Scalebar: 10 μm. **(B**&**C)** Immunostaining of MCF7 cells following treatment with the indicated concentrations of curcumin or garcinol for 24 hours. Vehicle control is also shown. Histone PTM-specific antibodies were used to reveal H3K18Ac, H4K16Ac and H3K9Ac levels in response to treatment. Scalebar: 10μm. **(D)** Western blots on whole cell extracts of MCF7 cells. Cell extracts were prepared following 24 hours culture in the presence of HAT inhibitors (or vehicle control) at the indicated concentrations. Specific antibodies were used to detect bulk levels of H3K18Ac, H4K16Ac and H3K9Ac. Densitometry measurements were performed using Image J software [21]. The level of each histone PTM in controls (vehicle only, normalised to a loading control) was set to 1. **(E)** Western blots showing bulk levels of H3K18Ac in whole cell extracts of U2OS and SaOS2 osteosarcoma cells following exposure to 10 μM or 20 μM curcumin or garcinol (as indicated in increasing scale). Actin loading controls are also shown, and the data were quantified by Image J as in (**D**).

We next compared the effects of curcumin and garcinol on bulk histone acetylation levels in proliferating MCF7 cells. Cells were treated with curcumin or garcinol at two sublethal doses (10 μM and 20 μM) for 24 hours, fixed and subjected to immunocytochemical staining with antibodies to detect *pan*-acetylated histones H3 or H4. As shown in Figure 2A, acetylated H3 was readily detected in the nuclei of controls, but stain intensity was reduced after exposure to curcumin, garcinol or LTK14, indicating H3 hypoacetylation, consistent with previous reports [18]. Similarly, acetyl-H4 staining appeared to decrease after exposure to 10 μM of the HAT inhibitors, but surprisingly was detected at similar intensity as control at the higher doses (20 μM) of curcumin or garcinol (Figure 2A). This unexpected effect appears to indicate differential and dose-dependent effects of these compounds on histone modifications in MCF7 cells.

To explore this further we next investigated how the acetylation status of specific histone N-terminal lysines is affected by HAT inhibitor treatments. Proliferating MCF7 cells were treated with inhibitor compounds as before, and assessed by immunocytochemistry and western blotting. Acetylation of H3K18, which is a substrate of CBP/p300 [22] was not inhibited by treatment with curcumin (up to a concentration of 20 μM) (Figure 2B&D). In contrast, garcinol treatment of MCF7 cells resulted in reduced staining with the H3K18Ac antibody (Figure 2B) and decreased detection of H3K18Ac by western blotting as confirmed by densitometry analysis (Figure 2D). In contrast, bulk levels of H3K9Ac in MCF7 cells were not altered following exposure to curcumin or garcinol (Figure 2B&D). Garcinol also reduced H3K18 acetylation in the osteosarcoma cell lines, U2OS and SaOS2, whereas curcumin treatment resulted in increased detection of H3K18Ac (Figure 2E). Thus, inhibition of CBP/p300, or other HATs required for progression through S phase [22], may account for the growth arrest induced by garcinol, whereas curcumin-induced growth arrest appears to involve a distinct mechanism.

In common with other cancer cell lines [6] and breast tumours [7], MCF7 cells exhibit low levels of acetylated H4K16 by immunofluorescence staining (Figure 2B&C). However, bulk levels of H4K16Ac were increased after exposure to curcumin or garcinol as observed by immunofluorescence (Figure 2B&C) and western blotting (Figure 2D) consistent with the moderate increase in *pan*-acetyl H4 levels (Figure 2A). These results indicate that selected H3 and H4 PTMs are differentially affected by curcumin and garcinol.

### DNA damage signaling pathways are induced by garcinol

**Figure 3:**
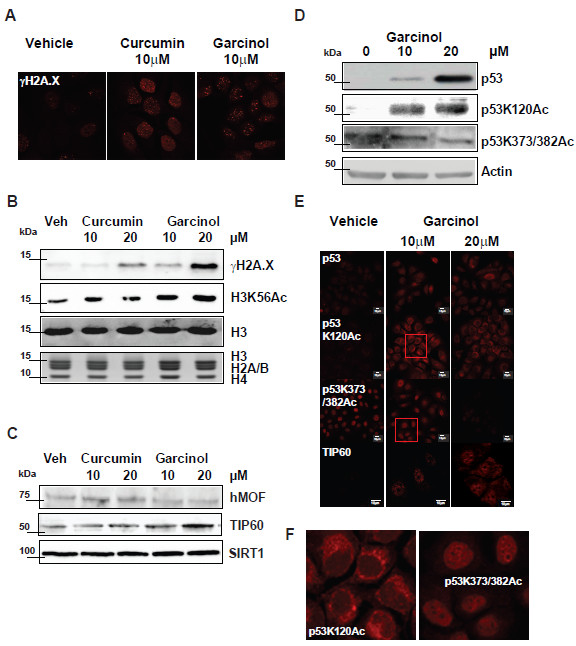
**Garcinol induces DNA repair pathways and alters p53 acetylation. (A)** Immunostaining of MCF7 cells for the DNA damage marker γH2A.X following treatment with curcumin, garcinol or vehicle for 24 hours. **(B)** Western blots of acid extracted histones prepared from MCF7 cells following treatment for 24 hours with HAT inhibitors at the indicated concentrations. Specific antibodies were used to reveal γH2A.X and H3K56Ac. Immunodetection of histone H3 and Coomassie staining of extracted histones are shown as loading controls. **(C)** Western blots showing the levels of hMOF, TIP60 and SIRT1 proteins in whole cell extracts of MCF7 cells following curcumin or garcinol treatment or control (DMSO). **(D)** Western blots detecting p53 expression levels and selected p53 acetylation PTMs (K373/382Ac or K120Ac) following garcinol treatment of MCF7 cells for 24 hours. (**E)** Immunostaining of MCF7 cells as treated in (**D**) showing the detection level and subcellular distribution of p53 and TIP60 proteins in MCF7 cells. Scalebar is 10 μm. **(F)** Higher magnification of boxed insets in (**E**) showing localisation of p53K120Ac and p53K373/382Ac proteins to the cytoplasm and nucleus, respectively.

The failure of garcinol-treated cells to complete S-phase, coupled with loss of H3K18 acetylation and enhanced acetylation of H4K16 prompted us to compare DNA damage signaling markers in HAT inhibitor-treated or control MCF7 cells. As shown in Figure 3A, curcumin and garcinol induced a dose-dependent increase in the number and intensity of nuclear γH2A.X foci, consistent with replication stress-associated DNA damage [23]. This was confirmed by western blotting, with a strong increase in γH2A.X phosphorylation observed after exposure to garcinol (Figure 3B). Garcinol also increased the levels of another DNA damage associated histone PTM, i.e. H3K56Ac (Figure 3B). Thus, inhibition of cell proliferation by garcinol is accompanied by a DNA damage signaling response in MCF7 cells.

### Garcinol alters expression and acetylation of tumour suppressor p53

The transcription factor p53 is an important regulator of cell fate decisions that also shows enhanced expression in response to DNA damage. Western blotting revealed a strong induction of p53 expression in MCF7 cells treated with garcinol (Figure 3D). Like histones, p53 function is regulated by lysine acetylation, which impacts on its function in transcription, DNA damage checkpoints and cell fate decisions. Acetylation of lysines in the C-terminal activation domain of p53 (K370, K372, K373, K381, K382) is mediated by CBP/p300 and PCAF and promotes transcriptional activation of p53 target genes [24]. However acetylation of p53 at K120 within the DNA binding domain can be catalysed by TIP60 in response to DNA damage, and has been implicated in activation of pro-apoptotic pathways both dependent on and independent of transcription [25-27]. Western blots further revealed that the K120-acetylated form of p53 is readily detected after garcinol treatment (Figure 3D), and correlated with increased expression of TIP60 (Figure 3C), whereas no changes in the levels of other chromatin regulators such as the MYST family HAT, hMOF or the deacetylase SIRT1, were observed (Figure 3C). Acetylation of the p53 C-terminal residues K373/382 was observed to be reduced by garcinol, consistent with its inhibitory effect on CBP/p300 activity (Figure 3D). Immunocytochemical staining revealed that the K120-acetylated form of p53 was only detected strongly after garcinol treatment, and was localised to the cytoplasm (Figure 3E&F). Consistent with the western data, increased expression of TIP60 was also observed in the garcinol-treated cells, but not controls (Figure 3E)*.* Taken together, these results suggest that garcinol has pleiotropic effects on breast cancer cells. Inhibition of CBP/p300 activity results in hypoacetylation of both histone and non-histone targets such as H3K18 and the C-terminus of p53, consistent with reduced gene transcription [18]. In addition, garcinol induces replication stress and DNA damage, resulting in upregulation of the DNA damage signals (γH2A.X, H4K16Ac, H3K56Ac) and associated proteins (TIP60, p53). The observed switch in p53 acetylation from C-terminus to DNA binding domain is consistent with altered functionality of p53 from transcription activator to growth arrest/apoptosis.

### SUV420H2 mediates H4K20 trimethylation induced by garcinol

**Figure 4:**
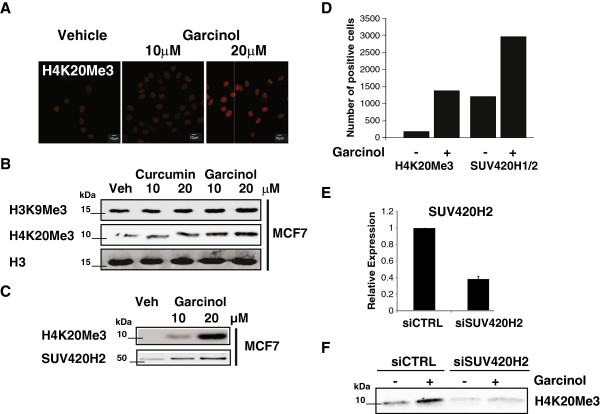
**Garcinol reprograms H4K20 trimethylation by inducing SUV420H2. (A)** Immunodetection of H4K20Me3 in MCF7 cells in response to garcinol treatment. Scalebar: 10 μm. **(B)** Western blots showing relative levels of H4K20Me3 and H3K9Me3 in acid-extracted histones prepared from MCF7 cells following treatment for 24 hours with HAT inhibitors at the indicated concentrations or control (DMSO). **(C)** Western blots showing induction of SUV420H2 and H4K20Me3 in MCF7 cells in response to garcinol exposure as in (**B**). **(D)** Quantitative analysis of the relative levels of SUV420H2 and H4K20Me3 in MCF7 cells in response to garcinol treatment as detected by flow cytometry. Cells were exposed to garcinol as in (**B**) and then fixed and permeabilised before incubation with primary and secondary (543 fluorophore-conjugated) antibodies. The data shown is the number of cells scoring positive for the indicated antigens from a total of 4000 scanned cells. **(E)** Relative levels of SUV420H2 transcripts in MCF7 cells at 24 hours post-transfection with siRNA duplexes targeting SUV420H2 (siSUV420H2) or a scramble control (siCTRL) siRNA mediated knockdown. **(F)** Western blots on MCF7 cell extracts following siRNA targeting as in (**E**) followed by exposure to garcinol (20 μM) for a further 24 hrs. The blot shows detection of H4K20Me3 levels following garcinol treatment in the control or SUV420H2 depleted cells.

In addition to effects on histone acetylation, we assessed whether garcinol might affect H4K20 trimethylation, as this PTM is reduced in cancer cell lines [6]. Immunostaining of control (vehicle-treated) cells indicated a relatively low detection level of this PTM in MCF7 cells. Unexpectedly, a strong dose-dependent enhancement of H4K20Me3 was detected after exposure of cells to garcinol for 24 hours (Figure 4A). Western blotting confirmed the garcinol-dependent induction of H4K20Me3, whereas no dramatic change in the level of trimethylated H3K9 was detected (Figure 4B). This induction of H4K20Me3 was unexpected as garcinol is not known to have direct effects on the activity of lysine methyltransferases, thus we reasoned that this might be due to an indirect mechanism, such as by affecting the expression of chromatin modifying enzymes.

Trimethylation of H4K20 in mammalian cells is catalysed by SUV420H2 [28,29]. As shown in Figure 4C, garcinol treatment of MCF7 cells resulted in a concomitant increase in both SUV420H2 and H4K20Me3. This was confirmed by flow cytometry/immunostaining of permeabilised cells, which detected an approximate 3-fold increase in the number of MCF7 cells expressing high levels of SUV420H2 protein, and an almost 10-fold increase in H4K20Me3 positive cells (Figure 4D). Similar induction of H4K20Me3 was observed in MCF7 cells treated with garcinol analogs (data not shown). These results indicate that increased SUV420H2 expression is likely to be responsible for the bulk increase in H4K20Me3 observed after garcinol treatment.

To test this hypothesis, siRNA duplexes were used to reduce the expression of SUV420H2. As shown in Figure 4E. SUV420H2 was successfully knocked down by a specific but not a control siRNA pool as measured by RT-qPCR. MCF7 cells were transfected with control or SUV420H2 siRNAs and subsequently treated with garcinol. Increased levels of H4K20Me3 following treatment with garcinol were observed in the control, however H4K20Me3 levels were strongly attenuated in the SUV420H2 knockdown (Figure 4F). Taken together our results indicate that SUV420H2 expression is induced after treatment of MCF7 cells with garcinol, and is responsible for the concomitant increase in trimethylation of H4K20. Thus, we conclude that garcinol treatment induces changes in expression levels of chromatin modifying enzymes in MCF7 cells, which drive changes in histone PTMs associated with DNA damage/repair responses and cell growth arrest.

## Discussion

Natural products are an important resource for the discovery of new leads for cancer therapies. Although garcinol has been shown to have cancer chemopreventive properties in animal models [30], its biological action remains poorly understood. While garcinol may have pleiotropic effects in cells due to its moderate antioxidant properties [31], the discovery that it can directly inhibit histone acetylation by p300 [16,17], indicates that it may impact directly on global histone modifications, and thus gene regulatory processes in tumour cells. However, significant gaps remain in our understanding of the biological effects of garcinol and related molecules on cell function.

A recent report showed that garcinol can block the proliferation of MCF7 breast cancer cells in culture [32]. Using concentrations of garcinol in excess of 25 μM, significant MCF7 cell apoptosis was observed [32]. In this study we confirmed the growth inhibitory effects of garcinol against MCF7 cells (Figure 1A), and established that garcinol is cytotoxic to these cells when used at concentrations in excess of 20 μM, inducing substantial loss of cell adherence and cell lysis (data not shown). As this precludes accurate measurement of the effects of garcinol on histone PTMs by western blot and immunocytochemical analyses, we performed experiments at subcytotoxic levels of garcinol (= or < 20 μM) to better understand its effects on lysine acetylation targets in MCF7 cells.

The reduced proliferation of MCF7 cells observed in MTT assays was confirmed by a dramatic decrease in the number of cells entering S-phase, as detected by BrdU incorporation in flow cytometry analyses (Figure 1B&C). However, the data suggested that the biological effects of curcumin and garcinol/LTK14 in these cells may be distinct. Curcumin-arrested cells showed an enhanced accumulation in G2/M, whereas cells treated with garcinol-related compounds arrested in G1. Similarly, HEPG2 cells have also been reported to arrest in G2/M after treatment with curcumin [33]. It is also worth noting that curcumin differed from garcinol in that it appeared to stimulate the growth of MCF7 cells at the lowest concentration tested (2 μM) (Figure 1A). This may be consistent with reports that low levels of curcumin can stimulate proliferation of other cell types, including neural progenitors [34] and 3T3-L1 preadipocytes [35]. Consistent with its anti-proliferative effects on a range of other cancer cell lines, curcumin blocked MCF7 cell growth at higher doses (10-20 μM) (Figure 1A). These results highlight the importance of considering the bioavailability of HAT inhibitor compounds to select dose ranges that inhibit rather than promote the growth of malignant cells.

Targeting of histone modifying enzymes is an area of emerging interest in the development of anticancer drugs. Pan-inhibitors of deacetylases (HDACs) have shown promise in preclinical models and have entered clinical trials. The involvement of CBP, p300, MOZ and MORF genes in chromosomal translocations associated with leukaemia [36] suggests that inhibitors of acetyltransferase enzymes may also have cancer chemopreventive properties. However, little is known regarding the biological effects of currently available lysine acetyltransferase inhibitors, such as garcinol. Consistent with previous studies on other cell types [16-18], we have shown here that treatment of MCF7 cells with curcumin or garcinol can lead to a dose-dependent reduction in bulk levels of histone acetylation, as determined using pan-acetylH3 and pan-acetylH4 antibodies (Figure 2A). Remarkably however, these compounds were found to have differential effects on bulk levels of selected PTMs encountered in histones H3 and H4 (Figures 2, 3, 4). While curcumin had no obvious negative effect on H3K18 acetylation at the concentrations tested, garcinol treatment resulted in H3K18 hypoacetylation in three cancer cell lines tested (Figure 2B,D&E). In contrast, neither compound was found to substantially affect H3K9 acetylation (Figure 2B&D). Acetylation of H3K9 has been shown to be catalysed by GCN5 [37] which is insensitive to garcinol [17,18]. Interestingly, recent studies have shown that acetylation of H3K18 by CBP/p300 is required for the activation of S phase in quiescent fibroblast cells [22,38]. Thus, garcinol inhibition of CBP/p300-mediated acetylation of H3K18 may be a contributary factor in the failure of MCF7 cells to proceed through S phase.

Although pan-acetylation of H4 was observed to be reduced by both curcumin and garcinol at 10 μM, we noted that at 20 μM this inhibitory effect was not as clear (Figure 2A). This anomalous result suggested differential dose-dependent effects of HAT inhibitors on H4 acetylation, and highlights the disadvantage of using pan-acetyl H3/H4 antibodies in that effects on specific histone PTMs can be masked. However, as shown in Figure 2B&C, H4K16 acetylation, which is known to be reduced in cancer cell lines [6], was barely detectable in control MCF7 cells at the concentration of antibody used. However, acetylated H4K16 was readily detected after treatment with curcumin (Figure 2B) or garcinol (Figure 2B&C). Acetylation of H4K16 is normally established by hMOF [39,40] although in conditions of cell stress other HATs can target this modification, e.g. the DNA damage-associated TIP60. We did not detect any change in the expression levels of hMOF after treatment with garcinol, whereas TIP60 expression appeared to be elevated (Figure 3C&E). Thus increased expression of TIP60 or other HATs may account for the increase in H4K16 acetylation. Although we also attempted to knock down TIP60 transcripts using siRNA in garcinol-treated cells, we did not observe a reduction in TIP60 protein levels by western blotting over the time course of the experiment (data not shown), thus we were unable to establish definitively whether TIP60 is responsible for the observed increase in H4K16Ac.

The observed elevation of γH2A.X foci in MCF7 cells exposed to garcinol (Figure 3A&B) is consistent with an increased incidence of DNA double strand breaks, likely associated with replicative stress [23]. Interestingly, acetylation of H2A.X by TIP60 has been reported to be required for phosphorylation of H2A.X S139 in response to DNA damage [41,42]. TIP60 is also responsible for acetylation of the DNA binding domain of p53 at K120 [27]. Our observations that garcinol induces expression of p53 and TIP60 in MCF7 cells (Figure 3C-E), accompanied by increased acetylation of p53K120 and its accumulation in the cytoplasm (Figure 3E&F), suggests that TIP60 may drive this switch in p53 function. This is consistent with other studies revealing that p53K120 is acetylated by TIP60 and that this is important for the apoptotic functions of p53 in response to DNA damage [27]. It has also been shown that p53K120Ac is enriched in the cytoplasm and associated with mitochondria where it impacts on apoptotic pathways [26]. We conclude that the garcinol-induced blockade of CBP/p300 inhibits acetylation of the p53 C-terminus and coupled with upregulation of TIP60 or other HATs, is likely to promote an acetylation-mediated switch in p53 function.

A surprising observation in our study was that garcinol also impacts on histone methylation, specifically trimethylation of H4K20 (Figure 4A-C). We have shown that this is due to the induced expression of SUV420H2 (Figure 4C-F), one of the major enzymes targeting H4K20 for multiple methylation. Like H4K16Ac, H4K20Me3 has been implicated in the repair of DNA damage [11] and cell senescence [43], both PTMs impact on chromatin structure [44,45], and a recent study has demonstrated their interdependence in gene transcription [46]. However, the consequences of reduced incidence of H4K20Me3 and H4K16Ac in breast tumours [7] remains to be determined.

## Conclusion

Our study indicates that in addition to inhibition of CBP/p300 acetyltransferase activity, garcinol has multiple biological effects in cancer cells, including the activation of DNA damage signaling and the induction of chromatin regulators such as TIP60 and SUV420H2. Moreover, we have provided proof of principle that histone PTM signatures associated with cancer can be reprogrammed by the natural product garcinol, a dietary compound with a traditional use as a chemopreventive agent.

## Abbreviations

BrdU: Bromodeoxyuridine;BSA: Bovine serum albumin;CBP: CREB-binding protein;CREB: Cyclic AMP-response element-binding protein;DMSO: Dimethylsulfoxide;FITC: Fluorescein isothiocyanate;GCN5: General control of nitrogen metabolism 5;HAT: Histone acetyltransferase;HDAC: Histone deacetylase;MOF: Males absent on the first;MORF: Monocytic leukemia zinc finger protein-related factor;MOZ: Monocytic leukemia zinc finger protein;MTT: 3-(4,5-dimethylthiazol-2-yl)-2,5-di phenyltetrazolium bromide;O.D.: Optical density;PBS: Phosphate buffered saline;PCAF: p300/CBP-associated factor;SUV4-20H2: Suppressor of variegation 4–20 H2;siRNA: Small inhibitory RNA;TIP60: HIV Tat-interacting protein 60

## Competing interests

The authors declare that they have no competing interests.

## Author’ contributions

HMC, MKA & DMH conceived and designed experiments and prepared figures. HMC, MKA, MM, SED, AA, KBK, BY and GSW performed experiments and analysed data. KM and TKK prepared and provided unique research materials. HMC and DMH wrote the manuscript. MKA, GSW and TKK helped edit the manuscript, and the final version was approved by all authors.

## Authors’ information

HMC and MKA are equal first authors.

## Pre-publication history

The pre-publication history for this paper can be accessed here:

http://www.biomedcentral.com/1471-2407/13/37/prepub
